# A novel endovascular robotic-assisted system for endovascular aortic repair: first-in-human evaluation of practicability and safety

**DOI:** 10.1007/s00330-023-09810-x

**Published:** 2023-06-20

**Authors:** Chao Song, Shibo Xia, Lei Zhang, Kundong Wang, Haiyan Li, Wenying Guo, Longtu Zhu, Qingsheng Lu

**Affiliations:** 1 Department of Vascular Surgery, Shanghai Changhai Hospital, Naval Medical University, Shanghai, 200433 People’s Republic of China; 2 Department of Instrument Science and Engineering, Shanghai Jiao Tong University, Shanghai, 200240 People’s Republic of China

**Keywords:** Robotic surgical procedures, Robotics, Aortic aneurysm, Endovascular aneurysm repair

## Abstract

**Objectives:**

To assess the practicability and safety of a novel endovascular robotic system for performing endovascular aortic repair in human.

**Methods:**

A prospective observational study was conducted in 2021 with 6 months post-operative follow-up. Patients with aortic aneurysms and clinical indications for elective endovascular aortic repair were enrolled in the study. The novel developed robotic system is applicable for the majority of commercial devices and various types of endovascular surgeries. The primary endpoint was technical success without in-hospital major adverse events. Technical success was defined as the ability of the robotic system to complete all procedural steps based on procedural segments.

**Results:**

The first-in-human evaluation of robot-assisted endovascular aortic repair was performed in five patients. The primary endpoint was achieved in all patients (100%). There were no device- or procedure-related complications or no in-hospital major adverse events. The operation time and total blood loss in these cases were equal to those in the manual procedures. The radiation exposure of the surgeon was 96.5% lower than that in the traditional position while the radiation exposure of the patients was not significantly increased.

**Conclusions:**

Early clinical evaluation of the novel endovascular aortic repair in endovascular aortic repair demonstrated practicability, safety, and procedural effectiveness comparable to manual operation. In addition, the total radiation exposure of the operator was significantly lower than that of traditional procedures.

**Clinical relevance statement:**

This study applies a novel approach to perform the endovascular aortic repair in a more accurate and minimal-invasive way and lays the foundation for the perspective automation of the endovascular robotic system, which reflects a new paradigm for endovascular surgery.

**Key Points:**

*• This study is a first-in-human evaluation of a novel endovascular robotic system for endovascular aortic repair (EVAR).*

*• Our system might reduce the occupational risks associated with manual EVAR and contribute to achieving a higher degree of precision and control.*

*• Early evaluation of the endovascular robotic system demonstrated practicability, safety, and procedural effectiveness comparable to that of manual operation.*

**Supplementary information:**

The online version contains supplementary material available at 10.1007/s00330-023-09810-x.

## Introduction

In the past decades, endovascular treatment has become one of the primary alternatives for aortic aneurysm repair [1, 2]; however, this treatment poses the risk of potential hazards to both patients and operators [3]. To improve its therapeutic effect, the procedures that are performed are more difficult [4], and procedural duration is extended, which results in increased radiation exposure for patients and surgeons [5, 6]. In the case of surgeons, long durations of standing in a lead apron may lead to performance reduction, exhaustion, and even orthopedic injuries [7, 8]. Currently, various robotic-assisted systems are available for percutaneous coronary intervention and peripheral artery disease [9, 10]. The Magellan robotic catheter system has been used to facilitate vessel cannulation during fenestrated endovascular aortic repair (EVAR) [11]. But this robot is essentially a steerable catheter that can be remotely controlled to facilitate arterial navigation—it cannot successfully deploy the stent graft and cannulate the contralateral limb gate. Other endovascular robots, such as Corpath, are designed based on percutaneous coronary intervention technology, and cannot handle the over-the-wire (OTW) devices for endovascular aortic repair (EVAR). So there is no endovascular robot available of completing the entire EVAR procedure for clinical research. Additionally, the low accuracy and high device damage rate associated with rolling friction highlight the need for a more reliable design [12].

Recently, we developed a novel endovascular robotic system (ALLVAS, Aopeng Medical). This system can be controlled remotely, which aids in addressing procedural challenges, thus decreasing the risk of occupational hazards caused during EVAR [13]. The system can also enhance the accuracy and control of the interventional procedure [12]. Based on these advantages, we used this new endovascular robotic system to perform EVAR in patients for the first time.

This study aimed to estimate the safety and practicability of using this system in the delivery and manipulation of guidewires, catheters, balloons, and stent grafts in patients undergoing elective EVAR.

## Method

### Study design

This prospective investigation of the first-in-human application of a novel endovascular robotic system in the endovascular repair of aortic aneurysms was conducted from August to September 2021. Patients undergoing EVAR were recruited. Our professional team performed all the operations at Changhai Hospital (Shanghai, China). The study protocol was approved by the Ethics Committee of Changhai Hospital (CHEC2021-097), and all participants provided written informed consent. All relevant data are presented within the paper.

### Patient and public involvement

The patients and public were not involved in the design, conduct, reporting, or dissemination plans of our research.

### Study population

Patients with aortic aneurysm confirmed by computed tomography angiography (CTA) were enrolled in this study. According to recommendations in relevant guidelines and literature, the inclusion criteria were (1) asymptomatic fusiform aortic aneurysm with a maximal aortic diameter of at least 55 mm (both thoracic and abdominal), (2) female patient with aortic aneurysm between 50 and 55 mm, (3) saccular aortic aneurysm, and (4) rapid expansion of aortic aneurysms (> 10 mm/year or > 5 mm/6 months) [14-16]. The major exclusion criteria included (1) aneurysm rupture or impending rupture (discontinuity in calcification or hyperattenuating crescent sign in CT images), (2) aortic dissection or non-atherosclerotic aneurysm, (3) previous aortic surgery, (4) active infection, (5) femoral artery diameter ≤ 7 mm, (6) severe tortuosity or calcification of the iliac artery, and (7) hostile neck anatomy.

### Endovascular robotic system

**Fig. 1:**
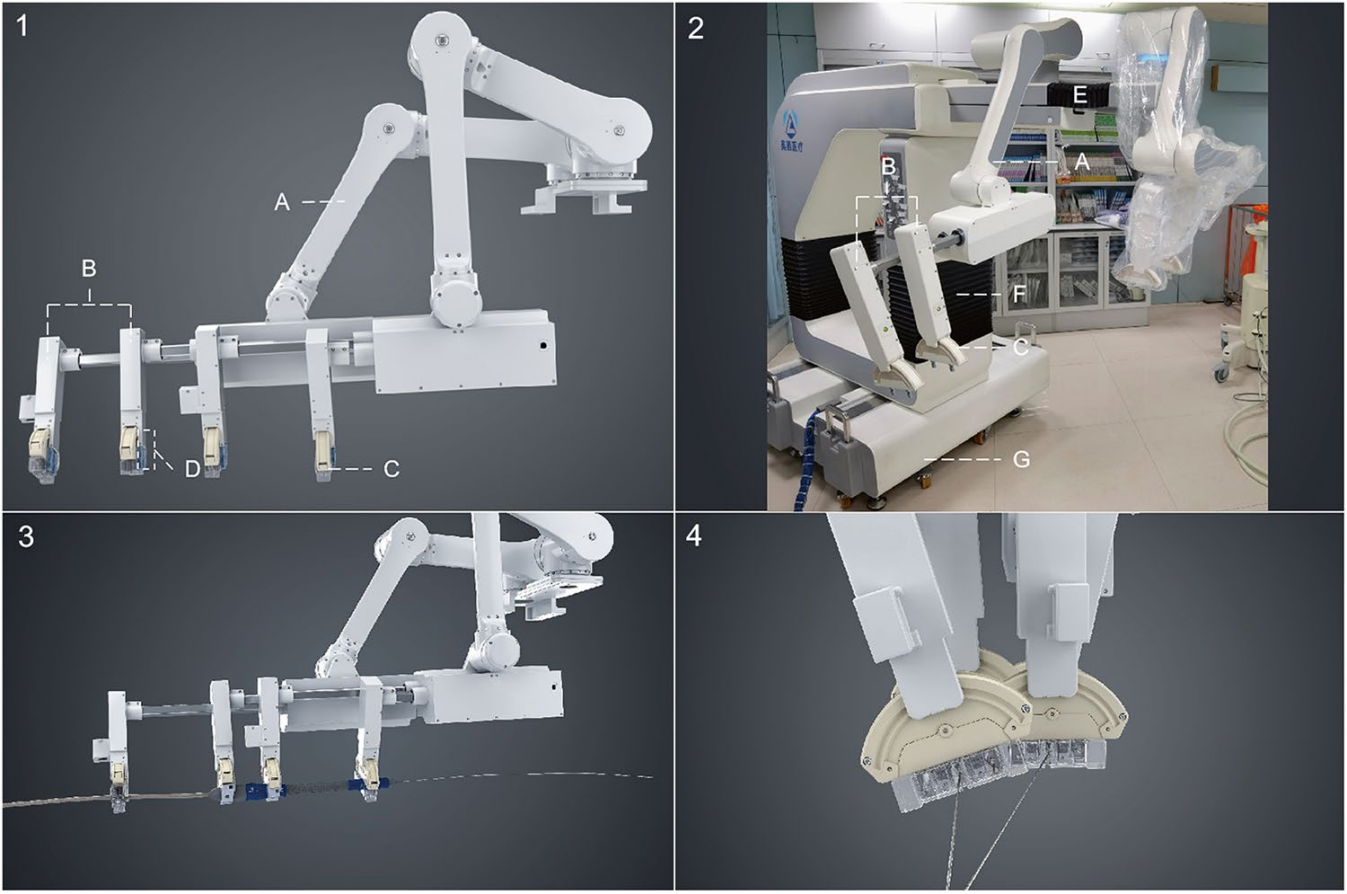
Functional unit of the endovascular robotic system. **1** Schematics of the functional unit. **2** Photograph of the functional unit. (A) Mechanical arm. (B) Manipulator. (C) Rotatable gripper. (D) Relay gripper. (E) Stretchable *Z*-axis motor. (F) Stretchable *Y*-axis motor. (G) Movable base. **3** Illustration of the compatibility of the endovascular robotic system with the over-the-wire system. **4** Illustration of the compatibility of the endovascular robotic system with the rapid exchange system

As a novel approach, the endovascular robotic system was developed for performing all endovascular procedures for aortic and peripheral artery diseases [13]. The system consists of two major parts: a functional unit and interventional console. The functional unit has universal architecture, making it compatible with market-leading devices, including both rapid-exchange and over-the-wire catheter systems. It can be adapted to any catheterization lab and does not require any specific equipment or technical changes prior to installation. The novel endovascular robotic system is designed with two groups of independent mechanical arms with three degrees of freedom so that the arms can independently perform a movement in three dimensions (Fig. 1). Each arm has two manipulators, and each manipulator has one gripper. The surgeon can control the long axial movement of the manipulators by operating the joystick with four manipulators, thereby clamping the endovascular devices. The long axial movement of the manipulators can be transformed into the movement of the devices.

**Fig. 2:**
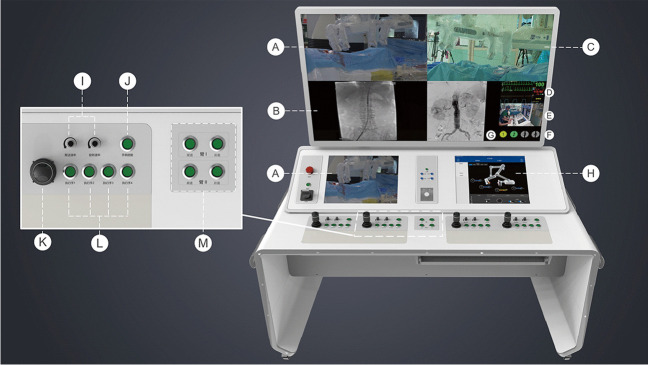
Interventional console of the endovascular robotic system. (**A**) Monitor of the functional unit (detailed view). (**B**) Real-time fluoroscopic image. (**C**) Monitor of functional unit (panoramic view). (**D**) ECG and hemodynamic monitor. (**E**) Monitor of the operator. (**F**) Motion state of the grippers. (**G**) Fluoroscopic image (Ref.). (**H**) Motion state of the functional unit. (**I**) Speed controller of manipulator movement and gripper rotation. (**J**) Joystick active button. (**K**) Joystick. (**L**) Manipulator selection button. (**M**) Controller of arm movements. Abbreviations: ECG, electrocardiogram

The interventional console is connected to the functional unit via a communication cable, which enables the operator to perform EVAR remotely from the console (Fig. 2). The interventional console, which is a radiation-shielded mobile workstation, can be placed anywhere indoors or outdoors. The operator manipulates the robotic system using four joysticks in the control console. The controlled manipulator can be switched arbitrarily through selection buttons. The operator can control any manipulator without moving his/her seat. The speed of movement and rotation can be modified by the speed controller, which facilitates the cannulation of the contralateral limb gate during EVAR. The real-time motion state of the functional unit is displayed on the monitor both in the detailed and panoramic views. Additionally, fluoroscopic images, electrocardiograms, and hemodynamic information are also displayed on the monitor inside the console, which can be visualized at a closer distance.

### EVAR procedure performed by endovascular robotic system

Before the intervention, two vascular surgeons independently analyzed the baseline CT images and suitability of the target lesion for EVAR. With this procedure, vascular access can be obtained using the Seldinger technique. Subsequently, the surgeon controlled the robotic system via the control console joysticks; the endovascular devices could then be advanced, retracted, and rotated.

**Fig. 3:**
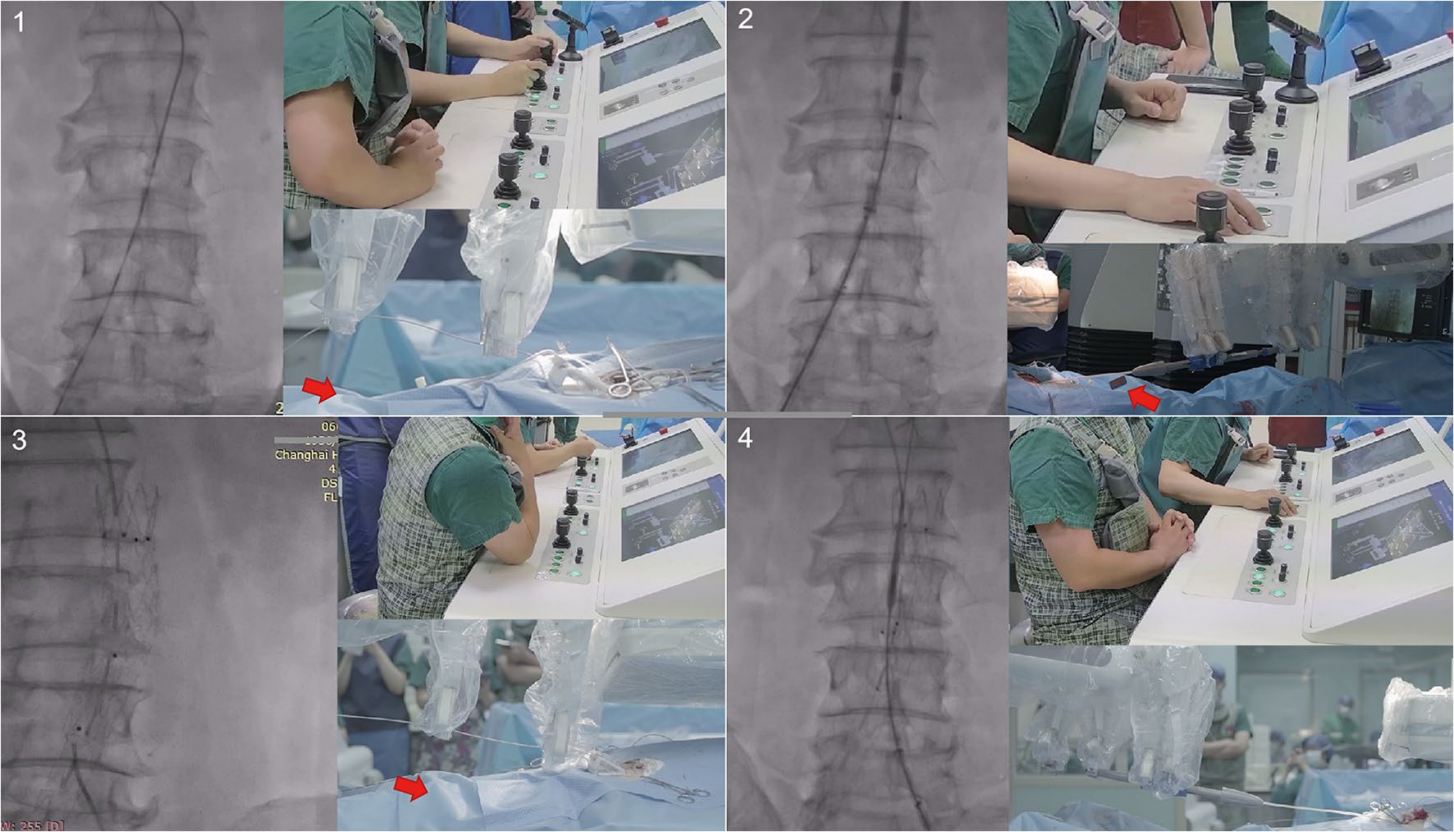
EVAR procedure performed by the endovascular robotic system. **1** Pigtail catheter advancement (red arrow: dosimeter location). **2** Stent graft release process (red arrow: dosimeter location). **3** Cannulation of the contralateral limb gate (red arrow: dosimeter location). **4** Stent graft release process (contralateral iliac limb). Abbreviations: EVAR, endovascular aortic repair

During EVAR of abdominal aortic aneurysm, the surgeon loaded a pigtail catheter into the robotic system and advanced the catheter along with the guidewire to the aorta above the target lesion to perform preoperative angiography. After angiography, a 0.035-inch extra-stiff guidewire (Lunderquist Extra Stiff Wire Guides, Cook Medical, Inc.) was automatically advanced into the thoracic aorta by the robot. The endovascular robotic system retracted the pigtail catheter and pushed the stent graft (Endurant, Medtronic Vascular, Inc.) into a predetermined position. The main body was deployed by the robot after repeated angiography to minimize the risk of renal artery occlusion. A multipurpose (MPA) catheter was used for the cannulation of the contralateral limb gate via a retrograde femoral artery approach, and the flexible design of the grippers guaranteed continuous rotation. The contralateral limb of the endograft was then introduced over another stiff guidewire using the robotic system. After the deployment of the contralateral iliac limb, the stent grafts were ballooned per the manufacturer’s instructions. Balloon insertion and retrieval were performed using the robotic system. A completion angiogram was obtained to verify the patency of the renal arteries and lack of gross endoleaks (Fig. 3). The device exchanges were performed by an assistant who was positioned behind a mobile lead shield during surgery. A detailed operation video is presented (see Video 1, which illustrates the key steps involved in robotic-assisted EVAR of abdominal aortic aneurysm).

The process for device insertion, deployment, and retrieval in EVAR of thoracic aortic aneurysm is analogous to that in EVAR of abdominal aortic aneurysm. Angiography was used to evaluate the efficacy of stent implantation and to rule out pertinent complications. A detailed operation video is presented (see Video 2, which illustrates the key steps involved in robotic-assisted EVAR of thoracic aortic aneurysm).

### Study endpoints

All patients were followed-up for major adverse cardiac events (MACEs) including death, myocardial infarction, or aneurysm rupture throughout their hospital stay and 6 months after discharge. The primary endpoint was technical success defined as the capability of the system to execute the procedure precisely and effectively; this was evaluated based on the number of procedural segments for the introduction and retrieval of all devices. Another endpoint was clinical success, which was identified by (1) accurate placement of the device and total exclusion of the aneurysm as well as (2) manipulation of the endovascular robotic system, deployment of a stent to the target lesion, and successful retraction of the delivery system without in-hospital and follow-up MACEs.

Radiation exposure to the operator at the console and procedure table was monitored using direct electronic dosimeters. The dosimeter on the procedure table was placed near the patient’s right leg and was shielded by a lead apron. The dosimeter was placed 30 cm away from the radiation source, which is the same as the distance between the operator and radiation source in manual EVAR.

The operation time included the setup of the robotic system and loading of the endovascular devices. Procedural attributes of the robotic system were recorded over time.

### Statistical analysis

Descriptive statistics were used to describe the patients’ baseline characteristics and in-hospital outcomes. Continuous variables were presented as the median (Q1, Q3) due to small numbers and categorical variables were shown as frequencies and percentages. No inferential statistical analysis was used.

This case series has been reported in line with the PROCESS Guideline [17].

## Result

Based on the inclusion and exclusion criteria, five patients underwent EVAR with the endovascular robotic system. Table 1 summarizes the patients’ demographic characteristics. There were four cases of abdominal aortic aneurysm and one case of thoracic aortic aneurysm. The average maximal diameter of the aortic aneurysm was 56.9 ± 2.16 mm. A hostile neck was defined as the presence of at least one of the following: neck length < 15 mm, neck diameter > 28 mm, angulation > 60°, mural thrombus/calcifications, and conical aortic neck [18]. There were no cases of hostile neck, which facilitated the EVAR procedure.

**Table 1:** Patient characteristics

Items	Patient 1	Patient 2	Patient 3	Patient 4	Patient 5	Median (Q1, Q3)/*n* (%)
Age, years	63	73	68	76	62	68 (63, 73)
Sex, *n* (%)	Male	Male	Male	Female	Male	4 (80)
Aneurysm location	Abdominal aorta	Thoracic aorta	Abdominal aorta	Abdominal aorta	Abdominal aorta	
Hypertension, *n* (%)	Y	Y	Y	Y	Y	5 (100)
Diabetes mellitus, *n* (%)	N	Y	N	N	N	1 (20)
Hyperlipidemia, *n* (%)	Y	N	N	Y	Y	3 (60)
COPD, *n* (%)	N	N	N	N	N	0 (0)
Prior myocardial infarction, *n* (%)	N	N	N	N	N	0 (0)
Prior stroke or TIA, *n* (%)	N	N	Y	N	N	1 (20)
Peripheral vascular disease, *n* (%)	N	N	N	N	N	0 (0)
Aneurysm morphology	Fusiform	Fusiform	Fusiform	Fusiform	Saccular	
Maximal diameter of aorta, mm	57.4	56.2	58.4	59.1	53.6	57.4 (56.2, 58.4)
Hostile neck of aneurysm, *n* (%)	N	N	N	N	N	0 (0)
Neck length, mm	32.1	45.3	19.2	31.2	39.3	32.1 (31.2, 39.3)
Neck diameter, mm	21.3	32.5	26.7	26.3	21.8	26.3 (21.8, 26.7)
Neck angulation, °	10.5	0	27.2	15.6	20.3	15.6 (10.5, 20.3)

*COPD*, chronic obstructive pulmonary disease; *Y*, yes; *N*, no; *TIA*, transient ischemic attack; *Q1*, lower quartile; *Q3*, upper quartile

For patient 1, the maximum diameter of the abdominal aortic aneurysm was 45.1 mm when measured 1 year ago, and the recent computed tomography (CT) scan confirmed that the maximum diameter of the aneurysm increased to 57.4 mm. The annual growth rate was > 10 mm/year, which was considered as rapid expansion.

For patient 4, this female patient with abdominal aortic aneurysm underwent an annual CT scan 6 months ago, and the maximum diameter of the aneurysm was 52 mm. Another CT scan was performed 1 week ago because of urolithiasis, and the maximum diameter increased to 59.1 mm. The annual growth rate was > 5 mm/6 months, which was considered as rapid expansion.

For patient 5, in this patient, the aneurysm morphology was saccular, and the maximum diameter of the aneurysm was 53.6 mm. Repair is generally recommended at a diameter of even < 55 mm.

All the procedures were performed via surgical cut-down femoral artery access. All patients received general anesthesia. All endovascular devices used in the procedure were commercially approved, and the therapeutic options were selected at the operator’s discretion. The robotic-assisted procedure included successful navigation and stent graft deployment in the patients. The guidewire proceeded smoothly, without dissection or perforation. The stent was successfully delivered to the lesion by using the novel robotic system. After stent graft deployment in all procedures, the stent delivery system was successfully retrieved into the sheath in all patients. Table 2 provides detailed information regarding these procedures. A conservative algorithm was adopted when operating on patient 1 so the operative time in patient 1 was relatively longer than the others.

**Table 2:** Detailed information regarding the procedure and in-hospital outcomes

Items	Patient 1	Patient 2	Patient 3	Patient 4	Patient 5	Median (Q1, Q3)/*n* (%)
Anesthesia	General	General	General	General	General	
Stents	Endurant	Valiant	Endurant	Endurant	Endurant	
Stent length (main body)	170	200	170	145	145	
Number of stents	3	1	3	3	2	
Hypogastric artery coverage	Right	-	-	Right	-	
Operative time, min	240	120	155	155	170	170 (145, 170)
Blood loss, ml	100	80	50	50	50	50 (50, 80)
Total contrast volume used, ml	170	80	130	110	110	110 (110, 130)
Fluoroscopy time, min	35	15	21	20	19	20 (19, 21)
Procedural radiation exposure, μGy						
Procedure table	150	40	110	125	90	110 (90, 125)
Operator	5	2	4	4	3	4 (3, 4)
Patient	2200	800	1435	1350	1200	1350 (1200, 1435)
Technical success	Y	Y	Y	Y	Y	
In-hospital death	-	-	-	-	-	
In-hospital MACEs	-	-	-	-	-	
Wound infection	-	-	-	-	-	

*MACE*, major adverse cardiac event; *Q1*, lower quartile; *Q3*, upper quartile; *Y*, yes

**Fig. 4:**
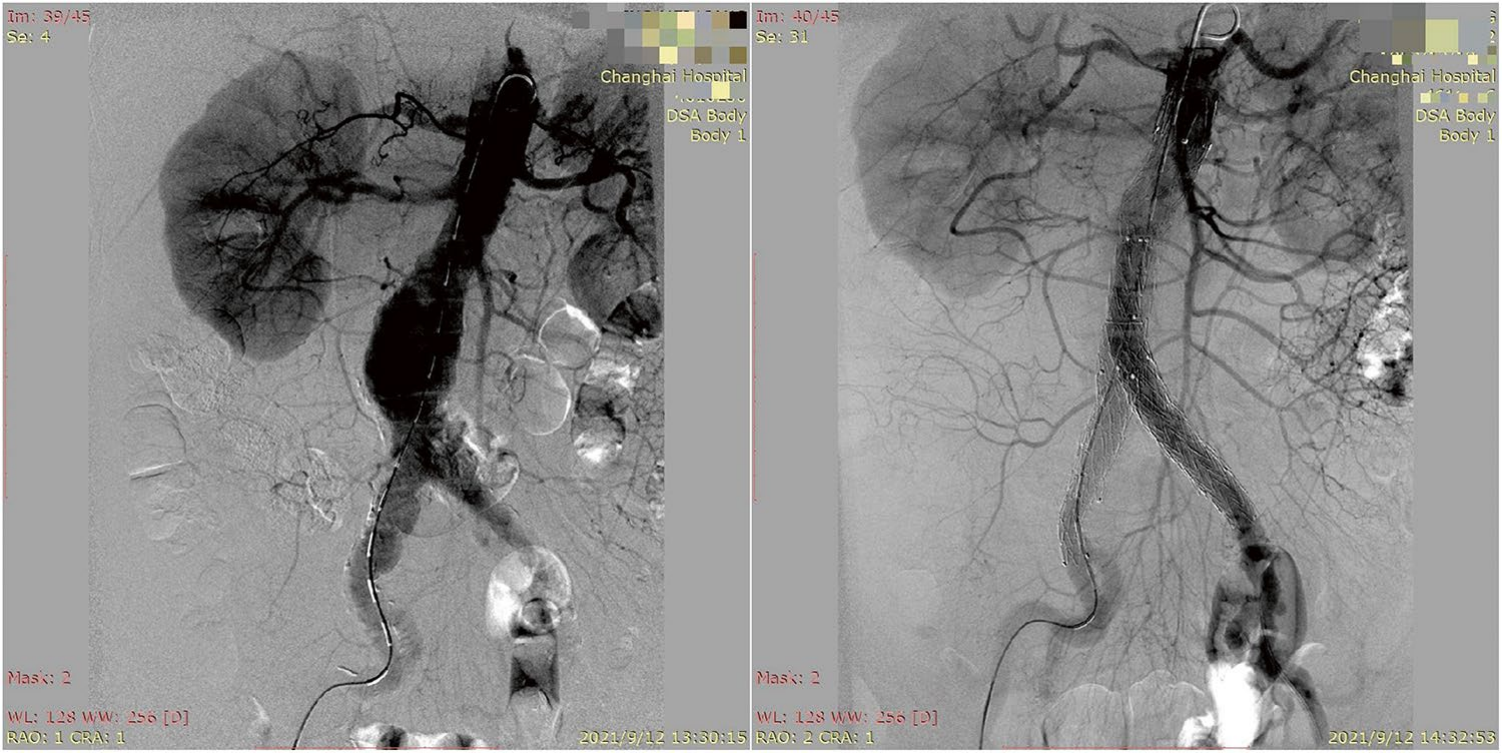
Endovascular repair of abdominal aortic aneurysm performed by the endovascular robotic system. (**A**) Preoperative angiography. (**B**) Postoperative angiography

Three of the four patients with abdominal aortic aneurysm received an extended iliac limb stent, and right hypogastric artery coverage was performed in two patients (50%). The operation duration did not exceed 3 h, except in the first case. The blood loss, total contrast volume used, and fluoroscopy time were consistent with those in the published literature [19, 20]. All procedures were completed using the robotic system, without any periprocedural complications. The angiograph of the treated lesions is shown in Fig. 4.

In summary, 100% of the interventional devices were successfully delivered and retrieved (overall technical success rate = 100%). No adverse clinical effects were associated with the endovascular robotic system. Thus, the primary endpoint, defined as technical success without in-hospital MACEs, was achieved in all five patients (100%). All patients were discharged within 72 h after surgery. The total radiation exposure of the operator at the console was 96.5% lower than that at the procedure table. The radiation exposure of the patients was not significantly increased when compared with traditional endovascular aortic repair [5]. At 6-month follow-up after the procedure, there was no incidence of MACEs.

## Discussion

This study involved a first-in-human experience with a novel endovascular robotic system and found that the system was safe and effective for the entire procedure of endovascular aortic repair. The endovascular robotic system was developed by our team and is now in the pre-commercial phase.

The research and development process associated with the currently available robotic systems are based on percutaneous coronary intervention (PCI). Consequently, the existing robotic systems are compatible with a rapid-exchange system, which limits the applicable outer diameter of the endovascular devices. These systems are controlled by the rotation of the manipulator, and they push or retract the catheter with the help of rolling friction applied through the rollers [21].

Unlike the currently available robotic-assisted system, this novel endovascular robotic system is designed with double V-shaped grippers, which can be adjusted arbitrarily according to the diameter of the endovascular devices [22, 23]. The double V-shaped grippers are adopted because roller design in other robotic systems inevitably leads to the relative sliding of the instrument and cannot execute self-expandable stent-graft deployment. Another reason for the double V-shaped grippers in the novel endovascular robotic system is to simulate manual gripping, which minimizes the additional risks associated with the modification of the current endovascular devices. The gripper used in the novel endovascular robotic system simulates manual operation to the greatest extent possible and increases stability during stent-graft deployment. As seen in the operation video, there was no stent migration during the procedure, which demonstrates the increased safety of the operation performed using the novel system. During this entire procedure, the demand for friction is not fixed in most cases; however, it is necessary to meet the requirement of minimum friction in order to reduce device damage. In contrast to rolling friction technology, the V-shaped gripper causes less damage to the hydrophilic coating of endovascular devices and is more beneficial in reducing the risk of vascular injury [24]. This design can also improve the efficiency of instrument replacement. Unlike in PCI treatment, different guidewires and catheters need to be replaced to accomplish angiography, arterial navigation, and other required actions in EVAR. The rapid opening and closing of the gripper can shorten the operation time of robotic-enhanced operations.

Another drawback in the application of the current robotic-assisted systems is that a considerable proportion of endovascular procedures are performed by surgeons and assistants. Existing robotic-assisted systems have only one set of robotic arms; therefore, they cannot meet current demands. One advantage of the novel endovascular robotic system is that it has two mechanical arms, each with two manipulators, which can be used to perform complex motions through the coordinated movement of the manipulators, including forward and backward movements and rotation of the manipulators (see Video 3, which demonstrates the entire process of wire advancement). With the help of these two mechanical arms, the catheter and stent can be moved forward and backward along the guidewire, the stent can be released, the system can be retracted freely, and finally, all the steps involved in EVAR can be completed. To prevent the superposition of torque in the direction of the axial deviation angle, which may damage the endovascular device and elevate the risk of iatrogenic injury, the axial state of the manipulators was controlled using software. The operating joystick controls the forward and backward movements and rotation of the manipulators. Furthermore, vascular access must be changed to implant the contralateral stent-graft limb during EVAR. With the novel endovascular robotic system, the position can be set freely via a stretchable *Z*-axis motor, whereas with other surgical robotic systems, the position can only be set after the alteration of the patient’s position, thereby increasing the surgical risk.

**Fig. 5:**
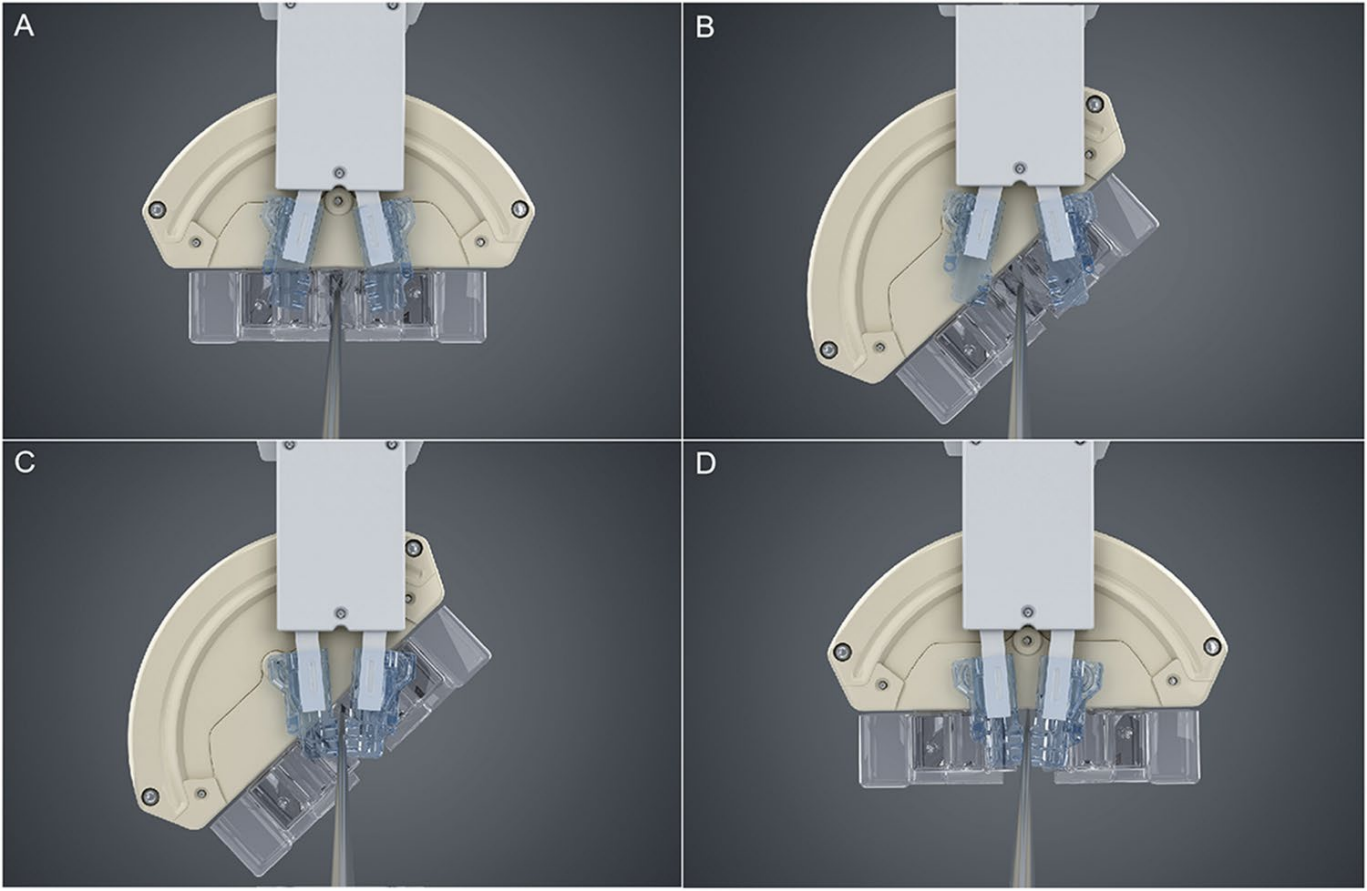
Progressive rotation of the grippers. Dashed red line represents the space between the rotatable grippers in different motion states. **A** Rotatable grippers clamp the wire, while relay gripper (red arrow) remains open. **B** Rotatable grippers rotate the wire, while relay gripper remains open. **C** Rotatable grippers release the wire, while relay gripper clamps the wire. **D** Rotatable grippers rotate back to the initial state, whereas relay grippers clamp the wire

For the cannulation of the contralateral limb gate using a retrograde femoral artery approach, the endovascular robotic system must rotate the guidewire and catheters continuously. On considering the potential risk of device entrapment during continuous rotation, we adopted the progressive rotation method using two groups of grippers. Briefly, the double V-shaped rotatable gripper rotates at an angle of 90° each time, and the third relay gripper relays to maintain the rotation angle until the rotatable gripper is reset and rotated again. Figure 5 demonstrates the progressive rotation (see Video 4, which illustrates the demo of the progressive rotation). Table 3 summarizes the main differences between this novel endovascular system and others.

**Table 3:** The comparison of different endovascular robotic systems

Country	Company	Product name	Compatible endovascular devices			Endovascular device delivery system	Actuation parts	Operations performed	Telecontrol
			Guide wires	Catheters	Stent grafts				
China	Aopeng	ALLVAS	All kinds of commercially available guide wires	All kinds of commercially available catheters	Stent delivery; can release	RX devices and OTW devices	Grippers	EVAR; TEVAR; PCI; PVI; NVI; VCFI	Y
USA	Hansen Medical	Sensei X Series	0.014 in, 0.018 in, 0.035 in	Artisan Extend Control Catheter	─	─	Tendon-driven guide catheter	PCI; CA; EVAR	Y
		Magellan	0.014 in, 0.018 in, 0.035 in	6F or 9F or 10F Magellan robotic catheter	─	─	Tendon-driven guide catheter	PCI; CA; EVAR; TEVAR	Y
USA	Catheter Precision	Amigo	─	Part of commercially available catheters	─	─	Friction wheels	CA	Y
USA	Stereotaxis	Genesis RMN	─	Magnetic drive catheter	─	─	Vdrive and magnetic control	CA	Y
USA	Corindus	CorPath 200	0.014 in	5-7F commercially available catheters	Stent delivery; cannot release	RX devices	Friction wheels	PVI; PCI	Y
		CorPath GRX	0.014 in	5-7F commercially available catheters	Stent delivery; cannot release	RX devices	Friction wheels	NVI; PVI; PCI	Y
France	Robocath	R-One	Commercially available guidewires	Commercially available catheters	Stent delivery; can release	RX devices	Friction wheels	PCI	Y
China	abrobo	RobEnt	Commercially available guidewires (0.035 in)	Commercially available catheters	Not reported	─	Gripping and rotational units	NVI	Y

*CA*, catheter ablation; *EVAR*, endovascular aortic repair; *NVI*, neurovascular intervention; *OTW devices*, over-the-wire devices; *PCI*, percutaneous coronary intervention; *PVI*, peripheral vascular intervention; *RX devices*, rapid exchange devices; *TEVAR*, thoracic endovascular aortic repair; *VCFI*, vena cava filter implantation; *Y*, yes

This rotation process was automated using software. The surgeon rotated the joystick only to issue rotation instructions and released it after achieving the desired angle. Currently, other robotic systems (e.g., RobEnt) also use gears for continuous rotation [25]. The advantage of our technique is that the rotation speed is faster than that of the other robotic systems, and the disassembly is less troublesome. In other robotic systems, the average loading time is 100 s, whereas the disassembling time is 30 s. By contrast, the replacement time of our endovascular robotic system and devices is within 10 s, which significantly shortens the operation time.

Studies have demonstrated that guidewire- and catheter-related iatrogenic dissection occurs during endovascular treatment in approximately 3.6% of cases. The risk of bleeding complications has ranged from 0.7 to 1.0% [26]. Although fluoroscopic guidance is likely necessary to improve the precision of stent graft deployment, the robotic-assisted system facilitates the positioning of the delivery system with a high degree of accuracy [27]. This endovascular robotic system was designed to enhance the precision and accuracy of endovascular procedures.

Proximal positioning is essential in EVAR, especially in patients with a short proximal landing zone. Through robotic technology, surgical efficiency can be increased or decreased such that the relatively larger movement generated by the surgeon through the handle can be transformed into subtle movements on the device. This function is performed through the regulation of the motor speed. Subsequently, the rotation speed was doubled, and the moving speed was decreased. This endovascular robotic system has the capability to precisely rotate (1° steps) the catheter and to accurately control (1-mm steps) the stent delivery system. The system effectively increased the directivity of the catheter tip, which facilitated the cannulation of the contralateral limb gate and reduced the risk of iatrogenic injury. Each surgeon can select the most comfortable operation mode to further reduce operative fatigue.

Additionally, the endovascular robotic system can be used for catheter advancement and exchange through preset automatic operation steps, which is particularly useful in debulking operations (e.g., thrombolytic drug spraying and thrombectomy). Through the standard automatic operating steps of the robot, effects of surgical inexperience on therapeutic outcomes can be avoided. This is beneficial for promoting the use of new devices. Robotic systems have been shown to reduce radiation exposure. A recent study suggested that the incidence of cataract-type eye opacities is three times higher in interventional cardiologists than in control subjects [28]. In our study, we confirmed a significant reduction (96.5%) in radiation exposure to the operator performing robotic EVAR.

The major limitations of the present study are the small sample size and selective population. Therefore, the novel endovascular robotic system needs further evaluation with larger study population. And it is necessary to find new solutions for patients with complicated conditions such as hostile neck, short landing zone, or ruptured aortic aneurysm. The endovascular robotic system is theoretically suitable for these circumstances due to its unique design. But implementation of complex operations like the fenestrated technique that may be applied in the procedure still needs preclinical assessment. Furthermore, remote control via 5G technology will also be applied to the robotic system in the future, which may contribute to alleviate the problem of insufficient medical resources in an underdeveloped area.

## Conclusions

Despite significant advances in endovascular technologies, the actual procedural methodology for EVAR has remained unaltered since the past 20 years. This novel endovascular robotic system might reduce the occupational risks associated with manual EVAR, in addition to contributing to a higher degree of precision and control during EVAR [29]. This study lays the foundation for the future development of efficient robotic platforms.

### Supplementary information

Below is the link to the electronic supplementary material.

Supplementary file1 (MP4 7169 KB)

Supplementary file2 (MP4 335 KB)

Supplementary file3 (MP4 7082 KB)

Supplementary file4 (MP4 5837 KB)

## Abbreviations

CTA: Computed tomography angiography
EVAR: Endovascular aortic repair
MACEs: Major adverse cardiac events
OTW: Over-the-wire
PCI: Percutaneous coronary intervention

## Declarations

### Guarantor

The scientific guarantor of this publication is Qingsheng Lu.

### Statistics and biometry

No complex statistical methods were necessary for this paper.

### Study subjects or cohorts overlap

No study subjects or cohorts have been previously reported.

### Methodology

• prospective

• experimental

• performed at one institution
